# Novel Variant of the *SLC4A1* Gene Associated with Hereditary Spherocytosis

**DOI:** 10.3390/biomedicines11030784

**Published:** 2023-03-05

**Authors:** Dżamila M. Bogusławska, Sebastian Kraszewski, Michał Skulski, Stanisław Potoczek, Kazimierz Kuliczkowski, Aleksander F. Sikorski

**Affiliations:** 1 Department of Biotechnology, Institute of Biological Sciences, University of Zielona Góra, Prof. Szafrana St. 1, 65-516 Zielona Góra, Poland; 2 Department of Biomedical Engineering, Wroclaw University of Science and Technology, Plac Grunwaldzki 13 (D-1), 50-377 Wroclaw, Poland; 3 Department of Cytobiochemistry, Faculty of Biotechnology, University of Wroclaw, F. Joliot-Curie 14a St., 50-383 Wroclaw, Poland; 4 Department and Clinic of Haematology, Blood Neoplasms and Bone Marrow Transplantation, Wroclaw Medical University, Wybrzeże L. Pasteura 4, 50-367 Wroclaw, Poland; 5 Silesian Park of Medical Technology Kardio-Med Silesia, ul. M. Curie-Skłodowskiej 10c, 41-800 Zabrze, Poland; 6 Research and Development Centre, Regional Specialist Hospital, Kamieńskiego 73a, 51-154 Wroclaw, Poland

**Keywords:** hereditary spherocytosis, anion exchanger 1, erythrocyte membrane protein, molecular dynamics simulation, whole exome sequencing

## Abstract

Hereditary spherocytosis (HS) refers to the group of the most frequently occurring non-immune hereditary hemolytic anemia in people of Caucasian central or northern European ancestry. HS is mainly associated with pathogenic variants of genes encoding defects in five membrane proteins, including anion exchanger 1 encoded by the *SLC4A1* gene. In this study, in a family affected with HS, we identified a hitherto unreported AE1 defect, variant p.G720W. The result of it is most likely the HS phenotype. Molecular dynamics simulation study of the AE1 transmembrane domain may indicate reasonable changes in AE1 domain structure, i.e., significant displacement of the tryptophan residue towards the membrane surface connected with possible changes in AE1 function. The WES analysis verified by classical sequencing in conjunction with biochemical analysis and molecular simulation studies shed light on the molecular mechanism underlying this case of hereditary spherocytosis, for which the newly discovered AE1 variant p.G720W seems crucial.

## 1. Introduction

Hereditary spherocytosis (HS) represents the most frequent non-immune hereditary hemolytic anemia in people of central or northern European ancestry, with a prevalence of 1:2000–5000 in Caucasians [1,2,3]. Although the disease is reported worldwide, it seems to be most common in Europe and North America. The lower prevalence of HS in people of other ethnic and racial backgrounds may be a consequence of the lack of comprehensive data from studies of other populations. An increasing number of reports in addition to early data concerning Japanese population band 4.2 variants have recently appeared from Korea and China [4,5,6,7,8]. They are characterized by the presence of spherocytes on the peripheral blood smear which are round and more fragile than normal erythrocytes. Reduced cellular deformability and mechanical stability are due to the absence of or more frequent defects in membrane proteins affecting membrane skeletal structure or membrane integral proteins’ connection to the membrane skeleton. These factors also result in a change in red cell shape caused by the decreased membrane surface area. All these changes lead to the shortened lifespan of erythrocytes, which makes them prone to hemolysis on their way through capillaries and phagocytosis mostly by the splenic macrophage system [9]. Splenectomy significantly increases the circulatory lifespan of cells. Loss of cohesion between the lipid bilayer and the cytoskeleton results in a release of skeleton-free lipid vesicles [3,10]. Microvesicles, containing anion exchanger 1, are released in case of spectrin, ankyrin, or protein 4.2 defects, which reduce the density of the cytoskeleton, destabilizing the overlying lipid bilayer. HS is associated with defects in several membrane proteins, including α- or β-spectrin, ankyrin, anion exchanger 1, and protein 4.2 (encoded by *SPTA1*, *SPTB*, *ANK1*, *SLC4A1*, and *EPB42* genes, respectively). A unique pathway is observed in the case of anion exchanger 1 defects, where the lipid-stabilizing effect is lost due to a deficiency of this transmembrane protein. In these cases, the released microvesicles are devoid of this exchanger [3,11].

The major membrane macro-complex of the erythrocytes, anchoring the spectrin-based membrane skeleton to the membrane bilayer containing integral proteins, is based on the interaction of ankyrin at one part of the molecule with two AE1 dimers and on the other part of the molecule with the β-spectrin constituting a subunit of the spectrin heterodimer or heterotetramer [12,13,14]. AE1 expression on the cell surface is facilitated by glycophorin A, a component of the AE1 complexes.

In the erythrocyte membrane, the AE1 transporter exists only in oligomeric (dimer/tetramer) form. Each AE1 monomer consists of an integral C-terminal membrane domain (mdAE1) and an N-terminal cytosolic domain (cdAE1) that anchors ankyrin-1 [15,16]. An anion exchanger is the attachment site for the membrane skeleton and plays a critical role in maintaining red cell hydration via catalyzing transmembrane Cl^−^/HCO_3_^−^ exchange, a crucial step for CO_2_ excretion [17]. Important data on the crystal structure of the protein were provided by Arakawa et al. [18] and Reithmeier et al. [15]. The membrane domain is responsible for the anion transport function of AE1 and each monomer comprises 14 transmembrane (TM) segments. Significantly, it creates two subdomains defined as the core (TMs 1–4 and 8–11, arranged in an inverted repeat) and the gate (TMs 5–7 and 12–14, containing the dimer interface.) [18]. Most helices contain more than 20 residues, which are minimally required to enclose the hydrophobic interior of the lipid bilayer. The exceptions are the TM3 and TM10 half-helixes, which face each other, giving the appearance of a continuous helix. A substrate binding site is located between the N-terminal ends of these helixes (TM3 and TM10) [15,18]. In contrast, TM9, which is a subject of the study described in this paper, is almost perpendicular to the membrane on the side of the core facing away from the gate.

Isoforms of AE1 are present in erythrocytes and kidneys, and their pathogenic variants are the cause of anemic disorders of the erythrocytes and also distal renal tubular acidosis in the kidney [19,20]. To date, 75 different variants of the *SLC4A1* gene associated with the HS phenotype, including 33 missense, 7 nonsense, 21 small deletions, 1 small insertion, 1 gross insertion, and 12 splicing mutations, have been reported (according to HGMD; http://www.hgmd.cf.ac.uk/; accessed on 28 January 2023). Other pathogenic variants of this gene are the cause of distal renal tubular acidosis (31) and blood group variation (22) and anemia (6) phenotypes. Hereditary spherocytosis is a clinically, biochemically, and genetically heterogeneous disorder in which the presenting phenotype is anemia, which can range from compensated to severe, jaundice, reticulocytosis, splenomegaly, gallstones, several spherocytes in a peripheral blood smear, and decreased erythrocyte osmotic resistance [1,21]. The largest group of HS patients present with a moderate manifestation of the disease [22]. The most common cause of spherocytosis is ankyrin deficiency, while 15–20% of cases are related to AE1.

Here, we report a previously unreported missense mutation (p.G720W) in the anion exchanger 1-encoding gene, which was detected using WES and verified by classical Sanger sequencing. It was correlated with the HS phenotype in a two-generation Polish family with autosomal dominant HS. Analysis of the red blood cell membrane profile showed a statistically significant decrease of AE1, which typically underlies HS. The newly discovered variant p.G720W of the *SLC4A1* gene is most likely the cause of clinical symptoms typical of hereditary spherocytosis.

## 2. Materials and Methods

### 2.1. Patients

Three patients from one family of Polish origin referred to Wroclaw Medical University, Department and Clinic of Haematology, Blood Neoplasms, and Bone Marrow Transplantation with a clinical diagnosis of HS were enrolled in our study. Patients with moderate symptoms of the disease were recruited: a father (AH137) and two sons (AH135, AH136), ages ranging from 39 to 66 years, who did not undergo splenectomy (Supplementary File S1: Figure S1.1). Other family members, including the mother of AH135 and AH136, reported as clinically asymptomatic, were inaccessible. The diagnosis of hereditary spherocytosis was essentially based on typical characteristics: anemia, spherocytes on peripheral blood smears, hypochromia and anisocytosis, splenomegaly, increased bilirubin, and reticulocytosis. The clinical manifestations in the studied patients were similar. This study was approved by the Ethics Committee of Wroclaw Medical University (protocol KB-199/2017). Informed consent was obtained from all patients before entering the protocol.

### 2.2. Red Blood Cell Membrane Isolation and SDS-PAGE Electrophoresis

RBC membranes were separated from peripheral blood according to Dodge et al. [23], with a small modification using a Ficoll-Paque PREMIUM density gradient (1.077 g/mL, GE Healthcare Bio-Science, Uppsala, Sweden) cushion to separate leukocytes and plasma, and the following were added to the final concentration: 100 μM PMSF (a serine protease inhibitor), 1 mM iodoacetamide (an inhibitor of cysteine protease), and a Protease Inhibitor Cocktail (in accordance with the recommendations of the manufacturer, Sigma), during hypotonic lysis and washes. The material was collected from three HS patients (AH135, AH136, AH137) and controls, i.e., three healthy volunteers (healthy individuals of Polish origin). Purified RBC ghosts were deep-frozen in liquid N_2_ and stored at −70 °C until they were subjected to sodium dodecyl sulphate polyacrylamide gel electrophoresis in the Laemmli buffer system [24]. Polyacrylamide gels (8% separating, 4.5% stacking, Rotiphorese, Roth) were stained with 0.012% Coomassie blue (R-250, Roth) in 10% ethanol and 5% acetic acid and destained in 10% ethanol and 5% acetic acid. Electrophoresis (SDS-PAGE) was performed at room temperature using a constant current of 25 mA in a running buffer (0.025 M Tris, 0.192 M glycine, pH 8.3 containing, 0.1% (*w*/*v*) SDS) until the tracking dye front reached the end of the polyacrylamide gel. Densitometric analysis was performed using ImageLab 6.0.0. software (Bio-Rad Laboratories, Inc., Hercules, CA, USA). The area under the densitometry curve allowed each band to be quantified. The amount of a particular protein was expressed as a percent of the total, where the sum of bands from α-spectrin to actin was considered as the total (1–5). The protein deficiency level was calculated in relation to controls. We used MS Excel software to process the densitometric data in this study, presented as mean ± standard deviation. Comparisons were performed using ANOVA test (Graph-Pad Prism software Inc., San Diego, CA, USA). Statistical significance was established at a *p* value < 0.05.

### 2.3. DNA and RNA Isolation

EDTA-preserved blood was collected from three patients (father AH137 and both sons AH135 and AH136) by venipuncture. For genomic DNA isolation from whole blood, the QIAamp DNA Blood Mini Kit was used (Qiagen, Hilden, Germany). Samples were stored at −20 °C until analysis. The miRNeasy Mini Kit was used for purification of total RNA from whole blood (Qiagen, Hilden, Germany). RNA samples were stored at −70 °C until use. Both isolation procedures were carried out in accordance with the manufacturer’s recommendations. Purified DNA and RNA concentration and quality were determined by absorbance in a Cary 60 UV spectrophotometer at 260 nm.

### 2.4. Whole-Exome Sequencing

Whole-exome sequencing and the bioinformatics analysis for detecting single-nucleotide variants (SNVs) and inserts/deletions were performed for each affected patient at the Heflin Center for Genomic Science Core Laboratories, University of Alabama at Birmingham (UAB), Birmingham, AL, USA. Whole blood DNA was subject to exome capture, performed using the Agilent SureSelect Human Clinical Research Exome (CRE) capture kit (Agilent Technologies, Inc., Santa Clara, CA, USA). The SureSelect Target Enrichment System (Agilent Technologies, Inc., Santa Clara, CA, USA) was used, followed by 100 bp paired-end sequencing on an Illumina NextSeq500 (Illumina, Inc., San Diego, CA, USA). Raw sequence reads were aligned to the reference human genome (human genome 19/GRCh37.13).

### 2.5. Whole-Exome Sequencing Data Analysis

Using the GEMINI (v0.19.1) software program, SNVs were annotated according to dbSNP. Subsequently, for further filtering, annotation, and interpretation of detected variants, the Ingenuity Variant Analysis plugin (IVA; QIAGEN, Redwood City, CA, USA) was applied. The clinical significance of selected changes was made according to the 1000 Genome Browser database (https://www.internationalgenome.org), HGMD (http://www.hgmd.cf.ac.uk/), ClinVar (http://www.ncbi.nlm.nih.gov/clinvar/), Online Mendelian Inheritance in Man (OMIM) (http://www.omim.org/), Single Nucleotide Polymorphism (dbSNP) (https://www.ncbi.nlm.nih.gov/snp/), GeneCards (https://www.genecards.org/), and The Universal Protein Resource (UniProt) (https://www.uniprot.org/) databases (each accessed on 20 December 2022) as well as literature data. The inclusion/exclusion criteria for the selection of variants with potential pathogenic significance were based on ACMG guidelines [25]. The exclusion criteria included, first of all, no correlation of the variant with the hemolytic anemia phenotype, a high frequency of occurrence of the variant in the population, and the variant should not be reported as pathogenic. The inclusion criteria were primarily confirmation of the change in all studied HS patients, a new/rare variant, a variant that may be predicted to be disruptive/damaging to the protein for which it codes, a variant of the gene encoding a protein whose defects have a proven relationship to the HS/hemolytic anemia phenotype, and a novel amino acid residue change.

### 2.6. Validation of Variants

Targeted genomic DNA and cDNA classical Sanger sequencing was used to verify potentially significant variants detected by whole-exome sequencing. Total blood RNA was transcribed into cDNA in the reverse-transcriptase reaction using the RevertAid First Strand cDNA Synthesis Kit (Thermo Fisher Scientific, Waltham, MA, USA). Sequences of all primers used in this work are listed in Table S1.1 (Supplement File S1). Synthesis of primers and sequencing using the Sanger method were carried out by Genomed S.A. (Warsaw, Poland).

### 2.7. Molecular Dynamics Simulations

Based on chain A of 4YZF pdb entry (https://www.rcsb.org/ accessed on 1 January 2023) two separate mdAE1 models were prepared, containing the p.G381 to p.D887 single chain terminated with GLYP and CTER patches, one with p.G720 (wild type) and one with p.W720 (mutant). Missing loops and further model compounds were modeled using the CHARMM-GUI online tool [26]. Each obtained mdAE1 model was separately embedded into a lipid membrane containing in upper leaflet 45 (16:0/18:1) phosphatidylcholine (POPC) and 90 (d18:1/16:0) sphingomyelin (PSM) molecules, whereas the lower leaflet was composed of 72 (16:0/18:1) phosphatidylethanolamine (POPE) and 48 (16:0/18:1) phosphatidylserine (POPS) molecules. The membrane was saturated with 87 cholesterol molecules distributed between the two leaflets in order to balance the surfaces of the two lipid leaflets (38/49), giving a 34% lipid fraction. Such an asymmetrical, cholesterol-rich lipid composition agrees with known and recent literature findings [27,28,29,30]. Protein was oriented into the membrane using PPM 2.0 Web Server [31]. Both models were neutralized and ionized using dissolved 0.15 M NaCl and 0.025 M KCHO_3_ according to [32]. Finally, the simulation box was completed by 30932 (for the p.G720 model) or 30666 (for the p.W720 model) TIP3P water molecules giving a simulation box of 10 nm^3^. Minimization was carried out according to the embedded CHARMM-GUI online tool protocol consisting of many steps of model relaxation, from solvent by lipids to the protein itself, using the steepest descent algorithm in the Verlet cut-off scheme. A molecular dynamics simulation was next run for 500 ns, with a time step of 4 fs, in the NPT ensemble (constant number of particles, pressure, and temperature) with a Berendsen thermostat and barostat using semi-isotropic coupling at T = 309.75 K, under GROMACS (version 2022.4) software with the CHARMM36 force field [33,34]. For visualization and analysis purposes, Visual Molecular Dynamics (VMD version 1.9.4a53) was used [35]. Ion permeability and conductive pore geometry were calculated using the HOLE program (https://www.holeprogram.org/ accessed on 1 January 2023).

## 3. Results

### 3.1. Hematological Parameters and Red Blood Cell Membrane Profile

**Figure 1:**
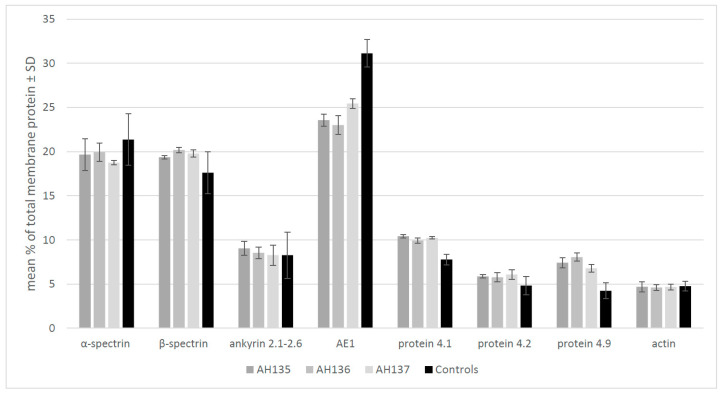
Erythrocyte membrane protein quantitative profile. Graph of individual erythrocyte membrane proteins for the averaged samples of AH family patients (AH135, AH136, AH137) in relation to the averaged control. Error bars represent standard deviation. Samples of all studied family members correspond to technical replicates (n = 3), while control samples correspond to biological replicates (n = 3, two technical replicates). Difference in AE1 content between the group of erythrocytes of six normal individuals was found to be statistically significant as calculated by ANOVA test, *p* < 0.0001. An Example of the original image of the Coomassie-stained SDS-polyacrylamide gel electropherogram and quantitative tracings are presented in Supplementary Figure S1.3.

Diagnosis was based on history, clinical findings, laboratory data, and morphological analysis of peripheral blood. Each studied proband exhibited similar clinical features resembling those of HS patients, namely, moderate anemia, bilirubinemia, and reticulocytosis. Basic hematological data for patients with HS are summarized in Table 1. SDS-PAGE allowed the identification and quantification of the major protein constituents of erythrocyte membrane (bands 1–5: α-spectrin, β-spectrin, ankyrin 1, anion exchanger 1, protein 4.1, protein 4.2, protein 4.9 (dematin, protein p55), and actin) and, thus, the identification of the protein membrane deficiency that underlies HS. Erythrocyte membrane proteins from AH family D patients are characterized by a substantial decrease in anion exchanger 1 content (Figure 1). In our analysis, their erythrocytes showed a statistically significant (*p* < 0.0001), over 22% decrease in this protein (Supplementary File S1: Figures S1.2 and S1.3). The ratios of ankyrin/AE1, spectrins/AE1, and protein 4.1/AE1 content (Table 2) confirmed the abovementioned conclusions. In addition, a substantial increase in band 4.9 protein content was observed.

### 3.2. Validation of Whole-Exome Sequencing Data

Whole-exome sequencing (WES) was carried out for each sample and included all available members of the studied family with symptoms of HS: the father (AH137) and both sons of AH137 (brothers AH135 and AH136). Quality control of WES raw reads is presented in Supplementary File S1: Table S1.2. Data verification was also performed using two bioinformatics tools: Ingenuity Variant Analysis and additionally GEMINI tools. We initially analyzed data based on phenotype–phenotype, phenotype–disease, and disease–gene networks constructed with QIAGEN. We used IVA-recommended filtering algorithms such as Common Variants, Predicted Deleterious, Phenotype Driven Ranking, and Biological Context, resulting in 5065/2456/42/5 variants, respectively. Of the five variants detected in the last type of filtering, there were four in the genes *SPTA1*, *SPTB*, and *SLC4A1*. Three of them were later verified by the Sanger method in the later stages of the research (rs41273519, rs200787781, and a new variant detected in the *SLC4A1* gene, p.G720W). One pathological change (rs1799945) in the *HFE* gene, detected in the heterozygous form in the AH137 patient, was excluded by us from further considerations due to the autosomal recessive type of inheritance observed in patients with hereditary hemochromatosis. However, importantly, only the variant p.G720W was present in all probands. In total, 45,560 nucleotide sequence changes present simultaneously in the genetic material in either of the probands from the AH family were detected, of which 893 known mutations/polymorphisms found in the Human Genome Mutation Database have been identified. Among them, two variants located in the *SLC4A1* gene (CM921015 (FP), rs5036) and *SPTA1* gene (CS995155 (DFP), rs28525570) proved to be particularly interesting in relation to HS phenotype. These two variants were confirmed in the genetic material of the patients using the Sanger method (Supplement File S1: Table S1.3). Literature data and information deposited in the ClinVar database indicate that both changes are rather mild and are detected in correlation with other variants as compound heterozygotes. In the next step, we targeted functional variants with a potentially high impact (e.g., missense, nonsense, frameshift, canonical +/−1 or two splice sites, and initiation codon). We primarily analyzed genes associated with clinical phenotypes of known hereditary hemolytic anemia according to recommendations of Russo et al. [36], including erythrocyte membranopathies, erythrocyte enzymopathies, and erythrocyte hemoglobinopathies. Over four hundred polymorphisms (404 known and 11 unknown variants) were detected in the genes included in the panel of 71 genes recommended for RBC pathologies by Russo et al. (for details, see Supplementary Files S2 and S3) [36].

**Figure 2:**
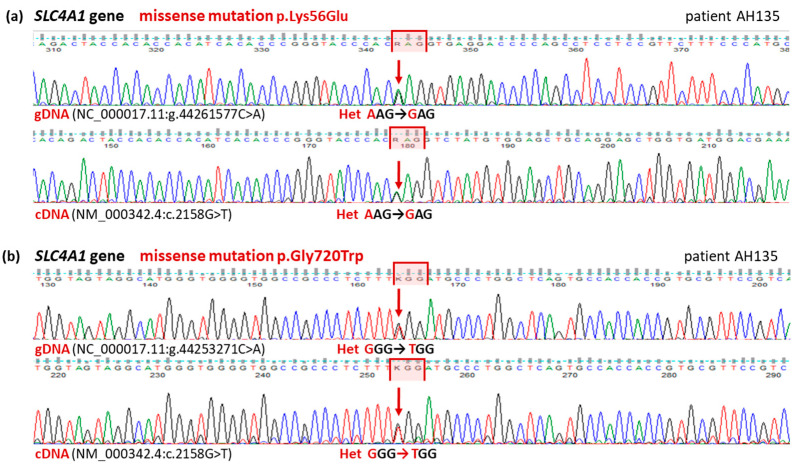
Two variants of the SLC4A1 gene detected in the patients with hereditary spherocytosis. Fragment of sequencing traces of the SLC4A1 gene in an affected patient (AH135). Both gDNA and cDNA sequence analysis revealed two heterozygous substitutions: (**a**) known, benign variant NC_000017.11:g.44261577T>C caused by the missense mutation p.K56E; (**b**) likely pathogenic variant NC_000017.11:g.44253271C>A caused by the missense mutation p.G720W.

Particularly interesting for us were five genes associated with clinical phenotypes of hereditary spherocytosis (*SPTA1*, *SPTA*, *SLC4A1*, *ANK1*, and *EPB42*). In these genes, we detected 78 variants (for details, see Supplementary File S3). Among these variants, according to the inclusion/exclusion criteria provided in the section Materials and Methods (Section 2.5), we selected six potentially crucial changes (including rs5036 and rs28525570, described above) in three genes (*SLC4A1*, *SPTB*, and *SPTA1*), and we verified the presence in the gDNA and cDNA (Supplementary File S1: Table S1.3). The selection criteria were parameters such as frequency and/or clinical significance (ClinVar), CADD Score, and SIFT Function Prediction. Two variants were confirmed only in individual patients: AH135 in the *SPTB* gene (NP_001342365.1:p.Arg2079His, rs200787781) and AH137 in the *SPTA1* gene (NP_003117.2:p.Arg2141Trp, rs41273519/ CM1611733 (DM)). Four other variants were confirmed in each patient, including two changes in the *SPTA1* gene (NM_003126.4:c.6531-12C>T, rs28525570/CS995155 (DFP) and NP_003117.2:p.Leu1858Val, rs3737515). Only in the case of the *SLC4A1* gene did the obtained results suggest potential significance for the phenotype (Figure 2). We detected two heterozygous missense mutations (one of them unknown) in all patients from the studied family: NP_000333.1:p.Lys56Glu (rs5036/CM921015 (FP)) and a new nucleotide change, NP_000333.1:p.Gly720Trp).

Both variants discovered through WES were automatically classified using Ingenuity Variant Analysis plugin results (IVA; QIAGEN, Redwood City, CA, USA) based on the American College of Medical Genetics and Genomics and the Association for Molecular Pathology Guidelines [25], using the known missense mutation **p.K56E** as a Benign variant, and the new variant **p.G720W** as a variant of Uncertain Significance: “Criteria for classifying **p.G720W** as Uncertain Significance variants: PM2: Absent from controls (or at extremely low frequency if recessive) in gnomAD [In these sources of population frequency data, this variant’s frequency is 0% or ≤0.001%] (Moderate); PP3: Multiple lines of computational evidence support a deleterious effect on the gene or gene product [CADD = 32.0] (Supporting)”. Variant Analysis plugin (QIAGEN, Redwood City, CA, USA) was predicted: for the **p.K56E variant**: CADD score 12.550, SIFT Function Prediction: Activating, SIFT Score: 1.0, and PolyPhen-2 Function Prediction: Benign; and for the **p.G720W variant**: CADD score 32.000, SIFT Function Prediction: Damaging, SIFT Score: 0.0, PolyPhen-2 Function Prediction: Probably Damaging and conservation phyloP *p*-value 2.168E-6 (for details, see Supplementary File S3).

### 3.3. Protein Modeling

**Figure 3:**
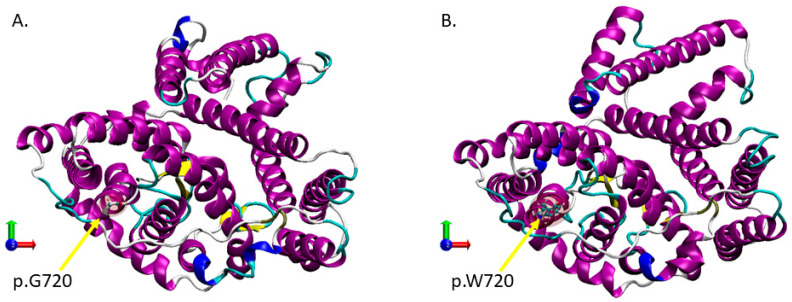
Illustration of the wild-type p.G720 protein model (**A**), and the mutant p.W720 protein model (**B**) after 500 ns of molecular dynamics simulation. Color code corresponds to protein secondary structure (purple—α-helix, blue—kink, white—turn, cyan—unstructured loop, yellow—β-sheet). Water, ions, lipids, and cholesterol molecules are not shown for clarity.

The starting configuration for the simulation of the p.G720 and p.W720 model systems was the transmembrane crystal structure protein (pdb 4YZF). For both models, two separate trajectories were generated. After the minimization protocol described above, each simulation was conducted for 500 ns. We first tested the stability of the structure of each protein with respect to the crystal structure used as a starting point. For both models, it was found that the RMSD (Supplementary File S4: Figure S4.1), the α-helix fraction (Supplementary File S4: Figure S4.2), and the fraction of the native contacts (Supplementary File S4: Figure S4.3) were quickly stabilized over the first 100–150 ns of the simulation trajectory. The stabilized value of the RMSD was about 1.0 Å higher for the mutant, i.e., 33% more unstable than for wild-type protein (calculated on the last 200 ns of simulation). Completely opposite behavior of the α-helix fraction was observed, amounting to 70% for both proteins, which is in the proper range for transmembrane proteins. On the other hand, the native contact fraction of the dynamically equilibrated structures decreased to about 88% for the p.G720 (wildtype), and to about 87% for the p.W720 (mutant), suggesting a slightly loose structure contact. Since there were not any signs of protein structural instability coming from parameters’ drift over simulation time, we proceeded to include a custom analysis of both models’ behavior in the comparative scheme. The location of residue 720 (Supplementary File S4: Figure S4.4) showed a permanent shift of 3.4 ± 0.9 Å of p.W720 towards the membrane surface, compared with the native p.G720. This important p.W720 displacement in the mutant led us to verification of the adjacent α-helix (p.S700-p.F719) length (Supplementary File S4: Figure S4.5). The results showed that the mean length of this α-helix in both models was actually the same, 28.3 ± 1.1Å for p.G720 compared to 28.6 ± 0.6 Å. The double reduction in fluctuations for the mutant is consistent with the results obtained for the overall fraction of α-helix (Supplementary File S4: Figure S4.2). Observed structural flattening for the mutant model (Figure 3), probably due to p.W720 displacement toward the membrane surface, compared with wild-type, led us to verify the conductive protein pore. The pore profile (Supplementary File S4: Figure S4.6) and its visualization (Supplementary File S4: Figure S4.7) show an important opening in favor of the p.W720 model. The HOLE program delivered theoretical ionic conductance equal to 2.3 pS/M for the p.G720 model and 260 pS/M for the p.W720 model.

## 4. Discussion

In our previous study, we reported new variants in the genes correlated with the hereditary hemolytic anemia phenotype including HS in a few Polish families [37,38,39,40,41]. In this report, we presented a Polish family with a novel missense mutation in the *SLC4A1* gene. In the studied family members, originally assigned as patients having hereditary spherocytosis, analysis of clinical symptoms and hematological data was consistent with WES data. RBC membrane protein gel electrophoresis demonstrated statistically significant changes in the membrane proteins (22%), including anion exchanger 1, thereby supporting the diagnosis of HS. The analysis showed that all studied patients presented a primary protein deficiency in anion exchanger 1 with an increased protein 4.9. As we did not find variants explaining any of the normal proteins included in this band, we suspect that proteolysis of AE1 protein could be the reason for this increase. According to the literature, in cases of hereditary spherocytosis correlated with *SLC4A1* gene mutations, a 20–35% loss of AE1 is usually observed [3,42,43]. In addition, in the studied family, we observe the typical autosomal dominant type of inheritance of AE1 defects (OMIM, #612653).

We decided to use the whole-exome sequencing method to identify the molecular mechanism that may be a direct cause for the clinical manifestation observed in patients from the studied family. Our WES-based analyses did not identify a single variant that is present in the HGMD database that could underlie anemia in the studied patients. The Ingenuity Variant Analysis plugin allowed us to filter the detected variants and limited the searches to variants correlated with red blood cell pathologies. Considering the clinical symptoms that indicate hereditary spherocytosis, we finally narrowed the searches to five genes associated with this phenotype. After analyzing 78 variants, we selected six potentially significant ones that we confirmed in the genetic material of patients using the Sanger method. Considering that the manifestation of clinical symptoms is similar in all patients, we classified two variants (rs200787781 in the *SPTB* gene and the *SPTA1* gene) occurring in individual family members as not significant or of low significance. We made a similar conclusion for two heterozygous truncating mutations of the *SPTA1* gene (rs28525570 and rs3737515). The first of these is a known mutation commonly referred to as the AlphaLELY allele [44,45]. This low-expression allele results in the expression of hereditary elliptocytosis (HE) when in trans with a pathogenic *SPTA1* variant. It is a consequence of overexpression of the α-subunit of spectrin. In patients with HS inheritance of *SPTA1*, defects are autosomal recessive (OMIM #270970). Based on the inheritance pattern in the studied family, we assume that the detected variants in the *SPTA1* gene most likely occur in cis.

Finally, we assumed that the two missense mutations of the *SLC4A1* gene (p K56E and p.G720W) detected in all probands most likely underlie the clinical HS in the family studied. The first known variant is not crucial to the identified defect. Memphis polymorphism (p.K56E) is the first reported variant of human erythrocyte anion exchanger 1 [22] and is detected in individuals of almost all ethnic groups with a frequency of about 0.05 (SNP NCBI, rs5036). As later proved, the defect causes a reduction in electrophoretic mobility of the 60-kDa fragment after external chymotrypsin digestion [46,47]. The defect is classified as benign/likely benign (ClinVar ID 17752) and is probably a target of recent natural selection [48]. On the other hand, Ideguchi et al. found that the transport rate of phosphoenolpyruvate in erythrocytes of heterozygotes was 10% lower than normal (in the case of homozygotes 20%) [49]. This variant is detected as coexisting with other variants in various red cell diseases such as blood group variation, Southeast Asian ovalocytosis, and hereditary spherocytosis [8,47,48,50]. However, the effect of the compound variant on the phenotypes has not been determined.

The second missense mutation (p.G720W) of the *SLC4A1* gene detected in the studied probands has not been described to date. Another missense mutation of the referenced glycine is classified by ClinVar as likely pathogenic (p.G720V; ClinVar ID1676958). The variant is associated with hereditary spherocytosis (OMIM #612653; SPHEROCYTOSIS, TYPE 4). Glycine is located in the core domain of the AE1 structure (TM10, discontinuously helical, TM10 Sequence: **G**MPWLSATTVRSVTHANA; UniProt ID P02730) [18].

The molecular dynamics simulation trajectories obtained for the wild-type and mutant mdAE1 showed that component helices did not change their structures in the p.G720W variant, but flattening of the tertiary structure of the mutant was observed. In addition, there was a reduction in all helices fluctuation, and in the α-helix adjacent to p.W720 (p.S700-p.F719), which should lead to stiffening of the mutant structure. Despite such structural changes in the protein, the thickness of the lipid membrane remained unchanged.

Although the shift of p.W720 to the outside of the membrane did not lead to deformation of the associated helix, it led to the widening of the protein structure in the plane of the membrane and consecutively led to a widening of the conducting pore. The consequence was a hundred-fold increase in conductivity that may lead to a loss of osmotic equilibrium, which in turn could lead to changes in mechanical properties of red blood cells and possibly the removal of these cells in the spleen.

Finally, we must note that the inclusion/exclusion criteria for the selection of variants with potential pathogenic significance (see Section 2.5) and the subsequent analyses of variants correlated with the hemolytic anemia/HS phenotype which we used during the segregation analyses contain some limitations. Used predefined data based on information deposited in biological databases and in the literature may not take into account undescribed and unverified facts for specific variants in genes that have not yet been linked to clinical symptoms or molecular mechanisms. It should also be mentioned that experimental verification of the functional effect of the observed mutation is not always relatively easy to perform, as our team did in the case of the ankyrin variant (p.L1340P) [37].

## 5. Conclusions

Application of whole exome sequencing together with previous data [36] allowed for precise detection of variant(s) in the case of hereditary spherocytosis. These changes in the genome and transcriptome were confirmed via standard Sanger sequencing. This may prove to be a strategy for detecting the molecular background of rare hereditary diseases.

Molecular dynamics simulations indicated the possibility of marked changes in the spatial conformation of the transmembrane domain of AE1 protein possibly leading to changes in function of this protein.

## Figures and Tables

**Table 1:** Hematological characteristics of studied family members. WBCs—white blood cells; RBC—red blood cells; Hb—hemoglobin; HCT—hematocrit; PLT—platelets; MCV—mean corpuscular volume; MCH—mean corpuscular hemoglobin; MCHC—mean corpuscular hemoglobin concentration; Ret—reticulocytes; IRF—immature reticulocyte fraction.

Laboratory Tests	Units	HS Patients			Mean ± SD	Reference Range
		AH135	AH136	AH137		
WBC	(G/L)	6.82	8.37	10.48	8.56 ± 1.84	4–10
RBC	(T/L)	3.7	3.59	4.31	3.87 ± 0.39	4.5–5.9
Hb	(mmol/L)	7.26	7.88	8.69	7.94 ± 0.72	8.69–11.17
HCT	(L/L)	0.33	0.35	0.4	0.36 ± 0.04	0.37–0.53
Total bilirubin	(µmol/L)	71.79	73.50	82.05	75.78 ± 5.49	3.42–20.51
Direct bilirubin	(µmol/L)	63.25	64.96	70.09	66.10 ± 3.56	<3.42
Indirect bilirubin	(µmol/L)	8.55	8.55	11.97	9.69 ± 1.97	0.00–8.55
PLT	(G/L)	183	148	203	178 ± 28	140–440
MCV	(fL)	89.5	97.8	92.6	93.30 ± 4.19	81–98
MCH	(fmol)	1.96	2.18	2.02	2.05 ± 0.11	1.61–2.11
MCHC	(mmol/L)	21.91	22.46	21.75	22.04 ± 0.37	19.24–22.96
Ret	(fraction)	0.0859	0.0942	0.084	0.0880 ± 0.01	0.005–0.015
IRF	(fraction)	0.164	0.207	0.188	0.186 ± 0.02	0.02–0.18

**Table 2:** Ratios of particular membrane protein contents. The analysis includes proteins whose defects are correlated with hereditary spherocytosis. Data from Figure 1 were used for calculations.

Band	HS Patients			AH Family	Controls (n = 3)
	AH135	AH136	AH137		
ankyrin/AE1	0.38	0.37	0.32	0.36	0.27
spectrins/AE1	0.82	0.88	0.78	0.82	0.57
protein 4.2/protein 4.1	0.56	0.58	0.59	0.58	0.62
protein 4.1/AE1	0.44	0.43	0.40	0.42	0.25
spectrins/ankyrin	4.32	4.70	4.66	4.55	4.72

## Data Availability

All data generated or analyzed during this study are included in this article and its additional files. WES datasets used and/or analyzed during this study are available from the corresponding author on reasonable request.

## Supplementary Materials

The following supporting information can be downloaded at: https://www.mdpi.com/article/10.3390/biomedicines11030784/s1, Supplementary File S1: Supplementary figures and tables. This file contains supplementary material: Figure S1.1. Pedigree chart of the required family with hereditary spherocytosis. Figure S1.2. Statistically significant differences of the erythrocyte membrane proteins for averaged samples of AH family patients in relation to averaged control. Figure S1.3. Analysis of erythrocyte membrane proteins. A representative SDS-PAGE gel image used for the tests for (a) samples of AH family patients and (c) controls (healthy individuals). Quantification of the erythrocyte membrane proteins in SDS-PAGE using densitometry. Examples of lane profile and peak area determination for (b) AH136 patient and (d) control. Table S1.1. PCR primer sequences. Table S1.2. Quality control of WES raw reads. Table S1.3. Polymorphisms identified during the Sanger sequencing analysis of all of the tested genes in the subjects from the studied AH family. Supplementary File S2: Supplementary tables. Detailed data on the analyzed variants detected using WES (in the panel of 71 genes recommended for RBC pathologies by Russo et al.) and analyzed using the GEMINI (v0.19.1) software program. Supplementary File S3: Detailed data on the analyzed variants detected by WES (in the genes whose mutations result in HS) and analyzed using the Ingenuity Variant Analysis plugin (IVA; QIAGEN, CA, USA). Supplementary File S4: Figure S4.1. Protein backbone root mean square deviation (RMSD) with respect to the X-ray diffraction structure (pdb 4YZF). Figure S4.2. Protein secondary structure: a-helix content of the wildtype (p.G720 model—black) and the mutant (p.W720 model—red) proteins. Figure S4.3. Native contacts fraction of the wildtype (p.G720 model—black) and the mutant (p.W720 model—red) proteins. Figure S4.4. Residue 720 location over simulation time for the wildtype (p.G720 model—black) and the mutant (p.W720 model—wine) proteins. Figure S4.5. The length of the α-helix (S700-F719) adjacent to p.G720 (black) and to p.W720 (red) in corresponding molecular dynamics models over simulation time. Figure S4.6. Conductive pore radius of the wildtype (p.G720 model—black) and the mutant (p.W720 model—red) proteins after 500 ns simulations.
