# Supplementary material for: Novel Variant of the SLC4A1 Gene Associated with Hereditary Spherocytosis
Source: Biomedicines. 2023 Mar 5;11(3):784. doi: 10.3390/biomedicines11030784 (PMC10045460; doi:10.3390/biomedicines11030784)
Supplement: Supplementary file 1 [file biomedicines-11-00784-s001.zip › Boguslawska et al Biomedicines_Additional File S1_R1f.pdf]

<u>Additional File S1</u>

Supplementary Material for Article:

Novel variant of the *SLC4A1* gene associated with hereditary spherocytosis

Bogusławska et.al

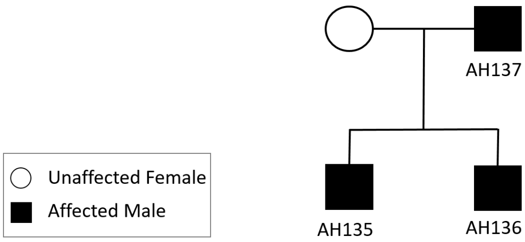

Figure S1.1 Pedigree chart of the required family with hereditary spherocytosis. Three studied family members (patients AH135, AH136, and AH137) and unaffected mother of AH135 and AH136 (not recruited for the studies).

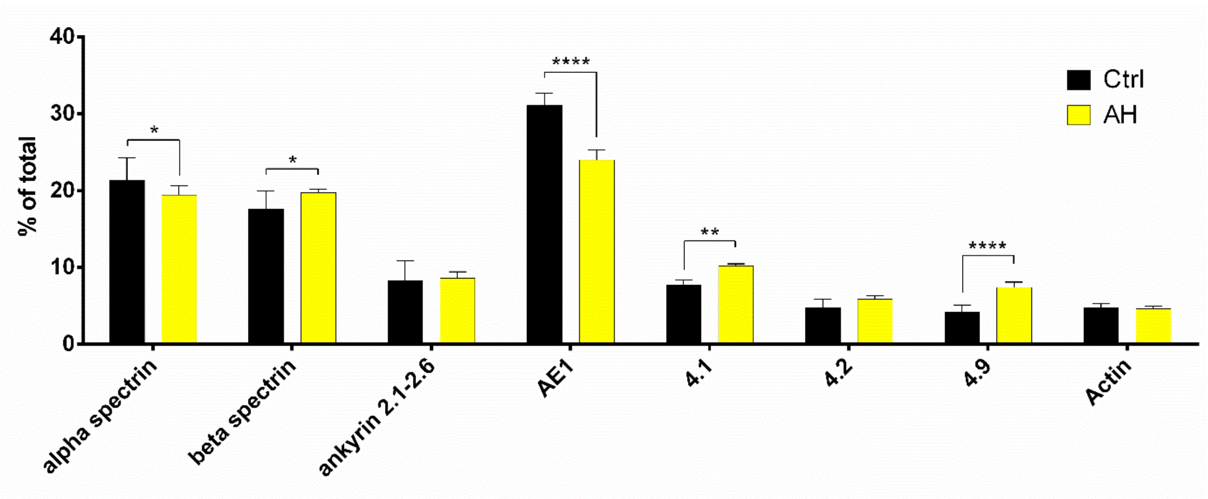

Figure S1.2 Statistically significant differences of the erythrocyte membrane proteins for averaged samples of AH family patients (AH135, AH136, AH137) in relation to averaged control (n=3). Error bars represent standard deviation. Statistical significance was accepted as a p-value of <0.05 was used.

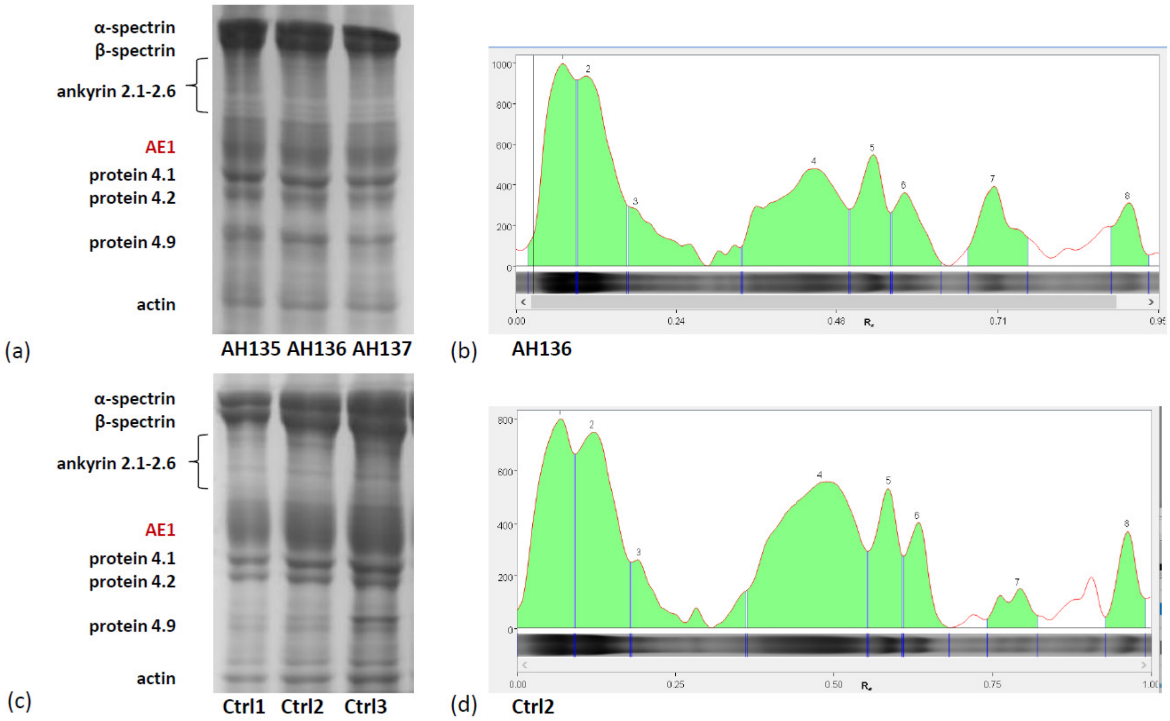

Figure S1.3 Analysis of erythrocyte membrane proteins. A representative **SDS-PAGE gel image used for the tests** for **(a)** samples of AH family patients (AH135, AH136, AH137) and **(c)** controls (healthy individuals). **Quantification of the erythrocyte membrane proteins in SDS-PAGE using densitometry.** Examples of lane profile and peak area determination for **(b)** AH136 patient and **(d)** control.

Table S1.1 PCR primer sequences.

Gene	Primer name	Forward primer sequence (5' to 3')	Reverse primer sequence (5' to 3')
SLC4A1 gDNA	p.G720W	GAGCTACAAGGACACCAAGTATGG	TGCTCTTTGACCTCCTGGATCTGG
SLC4A1 cDNA	p.G720W	GATACCTACACCCAGAAACTCTCG	GAAGCAAGATGCGGTCAAAGAGC
SLC4A1 gDNA	p.K56E	GGACGGTCAGGTCAATACTAACC	CCTTGTCAATCAGGTTATCTCTGC
SLC4A1 cDNA	p.K56E	CTCAGATCACCGTAGACAACTGG	TCTAGGAGGACAGTACCCTTGG
SPTB gDNA	p.R2079H	GGAACAAGTGTCAGGGCATGTGG	CAATGTCAGGTTTCTGATGAAAGGC
SPTA1 gDNA	p.R2141W	GAAGATAGGTTAGACTTGAGAGAGG	GATCCATTGAAGGAAGGTACTGGC
	c.6531-12C/T	GTGGACAGATTCATGTTTTGTGGC	GTCTCAGCACTAACTCTTTCTTAACG
	p.L1858V	GTCTGCCAATTTCATTGAATCAACCC	CTCACCTTATTTAGGATGTCTTCTCC

Table S1.2 Quality control of WES raw reads. Burrows-Wheeler Aligner (BWA) statistics summarize the percentage of uniquely mapped reads, the percentage of properly paired reads, the percentage of duplicated reads and the average depth for each individual and for combined lanes.

Sample	Total # raw reads	% mapped to genome	% properly paired	% duplication	Avg depth	LANE
AH135	28 331 901	28,240,607 (99.68%)	28,068,786 (99.07%)	9,45%	19,99	1
AH135	27 872 353	27,775,269 (99.65%)	27,601,003 (99.03%)	9,32%	19,68	2
AH135	28 573 580	28,484,744 (99.69%)	28,315,418 (99.10%)	9,54%	20,14	3
AH135	28 144 495	28,053,652 (99.68%)	27,886,441 (99.08%)	9,41%	19,86	4
**AH135**	91 432 483	91,282,005 (99.84%)	90,903,418 (99.42%)	23,84%	67,85	1-4
AH136	26 724 598	26,638,085 (99.68%)	26,472,570 (99.06%)	8,81%	18,98	1
AH136	26 268 025	26,176,052 (99.65%)	26,008,206 (99.01%)	8,71%	18,67	2
AH136	26 985 129	26,900,799 (99.69%)	26,737,957 (99.08%)	8,90%	19,14	3
AH136	26 526 693	26,440,980 (99.68%)	26,279,962 (99.07%)	8,80%	18,84	4
**AH136**	86 120 725	85,978,351 (99.83%)	85,614,163 (99.41%)	22,56%	64,97	1-4
AH137	23 820 819	23,747,217 (99.69%)	23,599,370 (99.07%)	7,85%	16,95	1
AH137	23 401 362	23,322,864 (99.66%)	23,172,581 (99.02%)	7,76%	16,67	2
AH137	23 978 902	23,908,031 (99.70%)	23,764,153 (99.10%)	7,93%	17,06	3
AH137	23 560 933	23,488,744 (99.69%)	23,345,966 (99.09%)	7,83%	16,78	4
**AH137**	76 014 746	75,888,321 (99.83%)	75,568,838 (99.41%)	20,43%	58,85	1-4

.

Table S1.3. Polymorphisms identified during the Sanger sequencing analysis of all of the tested genes in the subjects from the studied AH family (het – heterozygotic; abs – absence). *accessed on 21 January 2023 **According to ClinVar NCBI: Commonly referred to as the AlphaLELY allele, this variant is a low expression allele that results in partial skipping of exon 46 and expression of hereditary elliptocytosis when in trans with a pathogenic *SPTA1* variant (Wilmotte et al., 1999); This variant has been reported multiple times in association with hereditary elliptocytosis when present in trans with a pathogenic SPTA1 variant (Russo et al., 2018; Aggarwal et al., 2020; Suzuki et al., 2021) This variant is associated with the following publications: (PMID: 10192450, 29396846, 31602632, 32287101, 29484404, 30298500, 32581362).

Gene name	SNP Reference No. / HGMD	Change of nucleotide/ amino acid residue	Frequency of change* MAF/Minor Allele Count	Inheritance / patients (WES)			Clinical Significance ClinVar*	CADD Score/ SIFT Function Prediction
				AH135	AH136	AH137		
SLC4A1	new	NC_000017.11:g.44253271C>A<br>NM_000342.4:c.2158G>T<br>**NP_000333.1:p.Gly720Trp**	None	het	het	het	None	32.000/ Damaging
	rs5036/ CM921015 (FP)	NC_000017.11:g.44261577T>C<br>NM_000342.4:c.166A>G<br>**NP_000333.1:p.Lys56Glu**	C=0.029842 (9246/309836, ALFA)<br>C=0.058211 (15408/264690, TOPMED)<br>C=0.050016 (7010/140156, GnomAD)<br>C=0.042433 (5151/121392, ExAC)<br>C=0.0181 (81/4480, Estonian)	het	het	het	Likely benign	12.550/ Activating
SPTB	rs200787781	NC_000014.8:g.65234054C>T<br>NM_001355436.2:c.6236G>A<br>**NP_001342365.1:p.Arg2079His**	T=0.000146 (22/150918, ALFA)<br>T=0.000079 (21/264690, TOPMED)<br>T=0.000071 (10/140244, GnomAD)<br>T=0.000066 (8/121376, ExAC)<br>T=0.0002 (1/4480, Estonian)	het	abs	abs	Uncertain significance	23.600/ Damaging
SPTA1	rs41273519/ CM1611733 (DM)	NC_000001.10:g.158589121G>A<br>NM_003126.4:c.6421C>T<br>**NP_003117.2:p.Arg2141Trp**	A=0.002539 (568/223726, ALFA)<br>A=0.0016 (8/5008, 1000G)<br>A=0.0036 (16/4480, Estonian)	abs	abs	het	Conflicting interpretations of pathogenicity:<br>Pathogenic(1);<br>Likely pathogenic(1);<br>Uncertain significance(6)	27.400/ Damaging
	rs28525570/ CS995155 (DFP)	NC_000001.10:g.158587858G>A<br>NM_003126.4:c.6531-12C>T	A=0.21386 (4906/22940, ALFA)<br>A=0.242461 (64177/264690, TOPMED)<br>A=0.254474 (35633/140026, GnomAD)<br>A=0.256450 (30935/120628, ExAC)<br>A=0.3248 (1455/4480, Estonian)	het	het	het	Conflicting interpretations of pathogenicity:<br>Pathogenic(1);<br>Likely pathogenic(1);<br>**Benign(2)**;**<br>Likely benign(3)	14.090/-
	rs3737515	NC_000001.10:g.158597507G>C<br>NM_003126.4:c.5572C>G<br>**NP_003117.2:p.Leu1858Val**	C=0.28263 (7900/27952, ALFA)<br>C=0.242408 (64163/264690, TOPMED)<br>C=0.254255 (35584/139954, GnomAD)<br>C=0.3246 (1454/4480, Estonian)	het	het	het	Conflicting interpretations of pathogenicity:<br>Pathogenic(1); Benign(9);<br>Likely benign(1)	22.800/ Damaging
