# Supplementary material for: Novel Variant of the SLC4A1 Gene Associated with Hereditary Spherocytosis
Source: Biomedicines. 2023 Mar 5;11(3):784. doi: 10.3390/biomedicines11030784 (PMC10045460; doi:10.3390/biomedicines11030784)
Supplement: Supplementary file 1 [file biomedicines-11-00784-s001.zip › Boguslawska et al Biomedicines_Additional File S4.pdf]

Additional File S4

Supplementary Material for Article:

Bogusławska et.al

Novel variant of the *SLC4A1* gene associated with hereditary spherocytosis

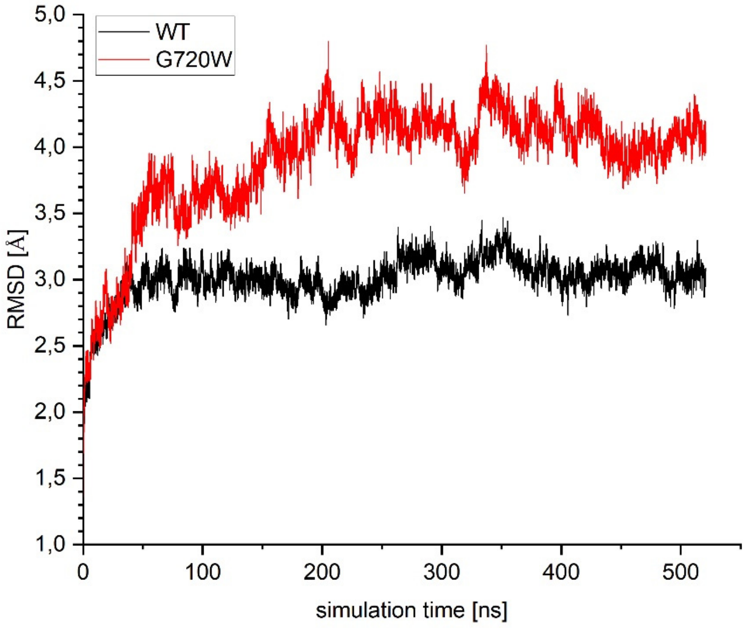

Figure S4.1. Protein backbone root means square deviation (RMSD) with respect to the X-ray diffraction structure (pdb 4YZF). The MD simulations were performed for both the wildtype (p.G720 model – black) and the mutant (p.W720 model - red) proteins at 309.75 K using CHARMM36 force field.

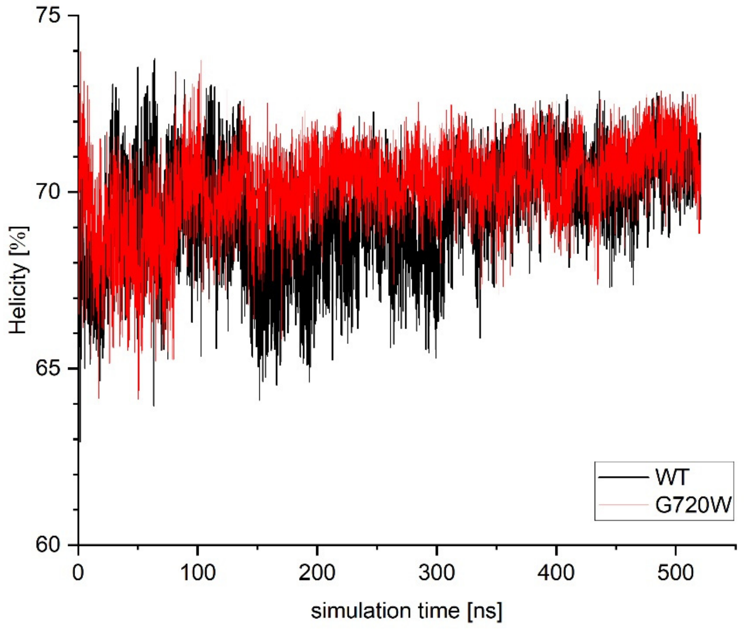

Figure S4.2. Protein secondary structure: a-helix content of the wildtype (p.G720 model – black) and the mutant (p.W720 model - red) proteins. The MD simulations were performed at 309.75 K using CHARMM36 force field.

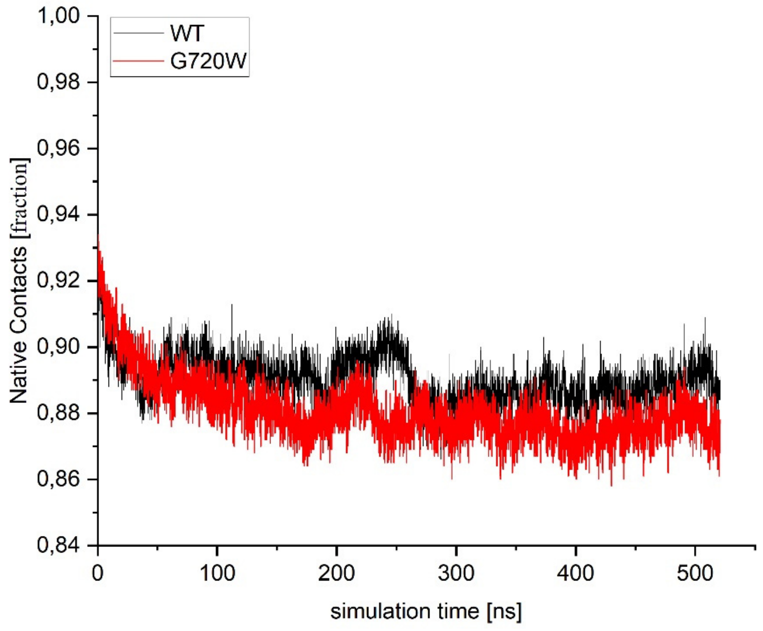

Figure S4.3. Native contacts fraction of the wildtype (p.G720 model – black) and the mutant (p.W720 model - red) proteins. The MD simulations were performed at 309.75 K using CHARMM36 force field.

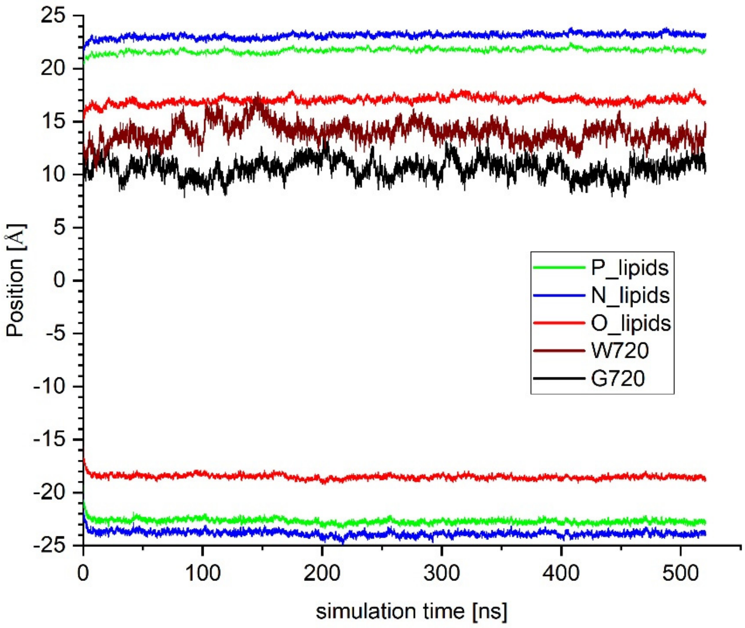

Figure S4.4. Residue 720 location over simulation time for the wildtype (p.G720 model – black) and the mutant (p.W720 model - wine) proteins. Mean position of phosphorous (green), nitrogen (blue), and carbonyl oxygen (red) lipid atoms are reported over simulation time for consistency. Lipid membrane in both models behaves identically, which allowed us to show lipid membrane fluctuations only from wildtype model for better clarity. Membrane center is positioned at zero.

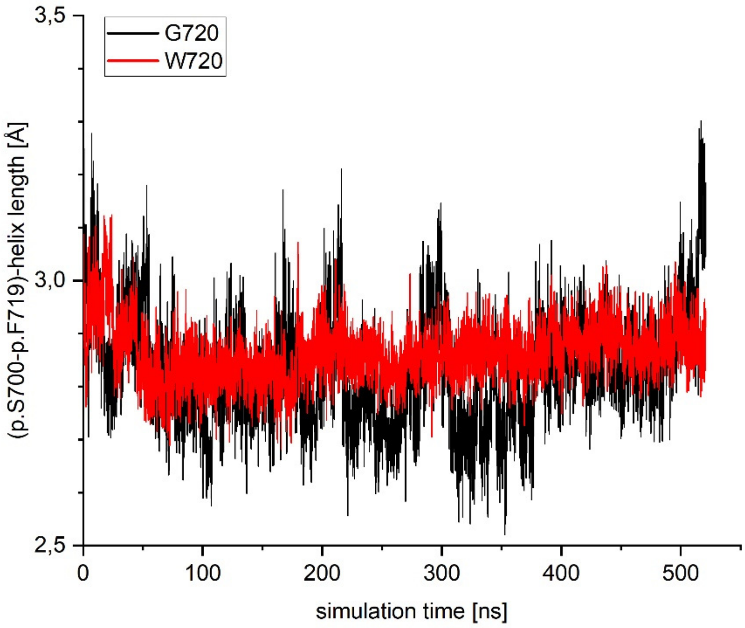

Figure S4.5. The length of the α-helix (S700-F719) adjacent to p.G720 (black) and to p.W720 (red) in corresponding Molecular Dynamics models over simulation time.

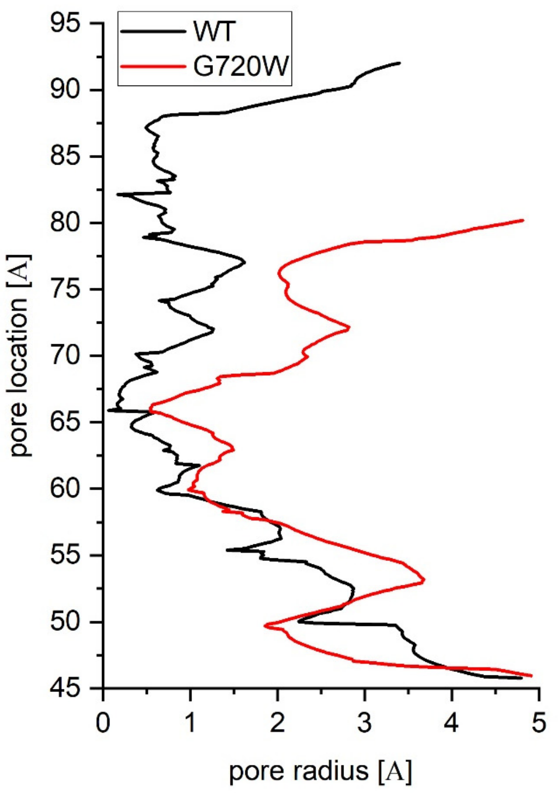

Figure S4.6. Conductive pore radius of the wildtype (p.G720 model – black) and the mutant (p.W720 model - red) proteins after 500ns simulations.

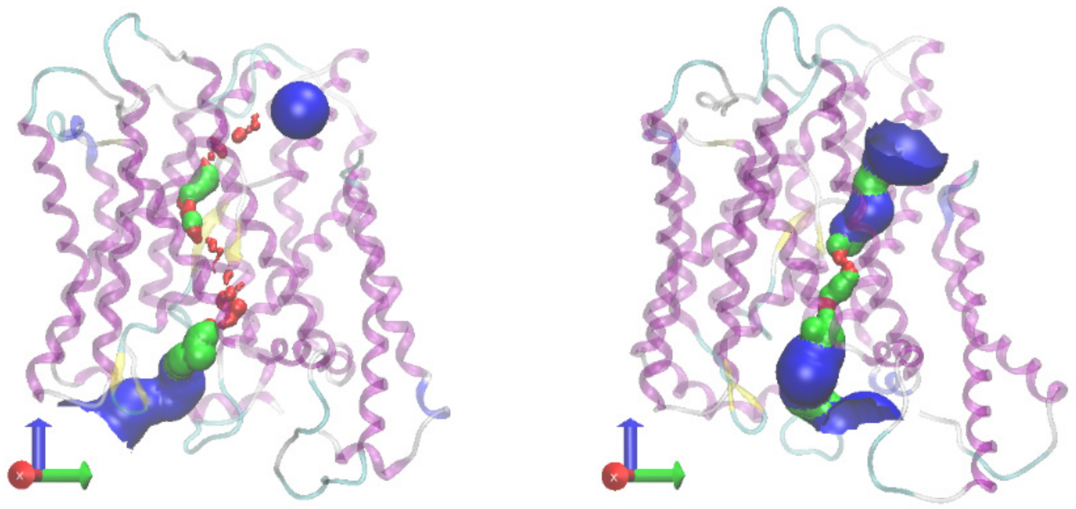

Figure S4.6. Visualization of conductive pore radius of the wildtype (p.G720 model – left) and the mutant (p.W720 model - right) proteins after 500ns simulations. Pore color corresponds to its radius from red – narrower to blue – larger. The same angle view was applied for better clarity.
